# Quality of Life and Depression Conditions of Women with Gestational Diabetes during Pregnancy and Postpartum Period

**DOI:** 10.1055/s-0043-1764494

**Published:** 2023-03-28

**Authors:** Reyhan Aydin Doğan, Nezihe Kizilkaya Beji

**Affiliations:** 1 Faculty of Health Sciences, Head of Midwifery Department, Karabuk University, Karabuk, Turkey; 2 Biruni University Faculty of Health Sciences, Istanbul, Turkey

**Keywords:** Gestational diabetes, Quality of life, Depression, Diabetes gestacional, Qualidade de vida, Depressão

## Abstract

**Objective** The study was conducted to determine the quality of life and depression of women with gestational diabetes during pregnancy and the postpartum period.

**Methods** 100 pregnant women with gestational diabetes and 100 healthy pregnant women were included in the present study. Data were obtained from pregnant women in their third trimester who agreed to take part in the study. The data was collected during the third trimester and six to eight weeks after the baby was born. The data were obtained by socio-demographic characteristics form, postpartum data collection form, the MOS 36 Item Short Form Health Survey and Center for Epidemiologic Studies Depression Scale (CESD).

**Results** The mean age of pregnant women with gestational diabetes in the study was the same as the average age of healthy pregnant women. The CESD score of pregnant women with gestational diabetes was 26,77 ± 4,85 while the corresponding score was 25,19 ± 4,43 for healthy women. Additionally, the score in the postpartum period was 32.47 ± 5.94 for pregnant women with gestational diabetes and 35.47 ± 8.33 for healthy pregnant women. CESD scores were found to be higher than the cut-off score of 16 in both groups, and the mean scores increased during the postpartum period.

**Conclusion** During the postpartum period, the quality of life of pregnant women with gestational diabetes was affected more negatively than healthy pregnant women. Depressive symptoms of women with both gestational diabetes and healthy pregnancy were found to be high in pregnancy and postpartum periods.

## Resumo

**Objetivo** O estudo foi realizado para determinar a qualidade de vida e depressão de mulheres com diabetes gestacional durante a gravidez e período pós-parto.

**Métodos** 100 gestantes com diabetes gestacional e 100 gestantes saudáveis incluídas no presente estudo. Os dados foram obtidos de mulheres grávidas no terceiro trimestre que concordaram em participar do estudo. Os dados foram coletados durante o terceiro trimestre e seis a oito semanas após o nascimento do bebê. Os dados foram obtidos por meio do formulário de características sociodemográficas formulário de coleta de dados pós-parto MOS 36 Item Short Form Health Survey e Center for Epidemiologic Studies Depression Scale (CESD).

**Resultados** A idade média das gestantes com diabetes gestacional no estudo foi igual à idade média das gestantes saudáveis. O escore CESD de gestantes com diabetes gestacional foi de 26 77 ± 4 85 enquanto o escore correspondente foi de 25 19 ± 4 43 para mulheres saudáveis. Além disso o escore no pós-parto foi de 32 47 ± 5 94 para gestantes com diabetes gestacional e 35 47 ± 8 33 para gestantes saudáveis. Os escores do CESD foram maiores do que o ponto de corte de 16 em ambos os grupos e os escores médios aumentaram durante o período pós-parto.

**Conclusão** Durante o período pós-parto a qualidade de vida de gestantes com diabetes gestacional foi mais afetada negativamente do que gestantes saudáveis. Os sintomas depressivos de mulheres com diabetes gestacional e gravidez saudável foram elevados na gravidez e nos períodos pós-parto.

## Introduction

Although pregnancy and childbirth are special and joyful times for women, changes in the body and the impression of being in less shape may lead to a decrease in self-esteem and depression in pregnant women. Other complaints, especially nausea and vomiting caused by the change of hormones, have a negative effect on the quality of life of pregnant women. 1

Apart from normal physiological factors, health problems in addition to pregnancy cause women to be more anxious and depressed. 2 These problems can be listed as concomitant hyperemesis gravidarum, preeclampsia, miscarriage threat, and gestational diabetes. 2 Gestational diabetes may occur during pregnancy. In the 1980's it is estimated to occur for 2–4% of all pregnancies in the world while it is given as 8.3% according to the 2019 world diabetes statistics. 3 4 During diabetes or pregnancy experiencing a serious disease such as the diagnosis of gestational diabetes mellitus (GDM) and the related implication that the baby may be affected adversely threaten the quality of life of the woman. 1 In the relevant literature no reports examining the quality of life of women and the presence of depression symptoms in women with gestational diabetes in Turkey could be found. On the other hand, studies indicating a significant relationship between the presence of chronic disease and postpartum depression in pregnant women and an increase in the frequency of depression in risky pregnancies have been reported. 2 5

The present study was planned to evaluate the quality of life and depression of women with gestational diabetes during pregnancy and postpartum periods.

## Methods

Pregnant women who applied to a public university hospital in Istanbul were included in the study. Ethics committee permission for the study was obtained from the ethics committee of Cerrahpaşa Medical Faculty Institution (Issue: B.30.İST.0.30.90.00 / 1594). Since the sample of the study was not similar before, it was planned to include 105 pregnant women with an effect size of 0.5, 95% confidence (1-α), and 95% test power (1-β) by double-sided *t* -test. In the study, 100 pregnant women agreed to participate in each group. Posthoc analysis was performed after the data were collected. In the study, when the posthoc analysis was examined by taking 100 cases in both groups; With 95% confidence (1-α), d = 0.407 effect size, the power of the test was 81.7% (1-β). Randomization of the study was made among the pregnant women who accepted the study, with single clinical hospitalization numbers as cases and doubles as controls. The case group included women with gestational diabetes who were in the last trimester of pregnancy. The diagnosis of GDM of the pregnant women in the case group was made by the diabetes polyclinic at the 24th week of pregnancy. The diagnostic criteria of the cases were confirmed by the diabetes polyclinic with the endocrine doctor and the obstetrician. The pregnant women in the case group were chosen from those who had been diagnosed with GDM by the 24th week of pregnancy and had been admitted to the obstetrics service when it was time to deliver. The International Association of Diabetes and Pregnancy Study Groups Consensus Panel was taken as a reference for the GDM diagnostic criteria of the cases. 6 Being pregnant with gestational diabetes, not having a dangerous pregnancy condition other than gestational diabetes, being in the last trimester of pregnancy, and accepting to participate in the study were the inclusion criteria for the case group. In addition, healthy pregnant women are included in the control group. The history and obstetric stories of the patients involved in the study were taken during their initial examinations. Healthy pregnant women must meet the following criteria: no chronic disease, not being pregnant at risk, being in the third trimester of pregnancy, and accepting to participate in the study. The identification of the patients (first name, last name, age), height, pre-pregnancy, number of pregnancies, gravida, parity, abortion, and living children were questioned.

In the study conditions, for the women in the case group, being pregnant with gestational diabetes, not having a risky pregnancy status other than gestational diabetes, being in the last trimester of pregnancy, and agreeing to participate in the research conditions were provided. For women in the control group, not having any chronic disease, not being pregnant at risk, being in the last trimester of pregnancy, and agreeing to participate in the research conditions were provided.

The data were collected by the socio-demographic data collection form, postpartum data collection form, The MOS 36 Item Short Form Health Survey and Center for Epidemiologic Studies Depression Scale (CESD) prepared by the researchers for the pregnant women who applied to the clinic and in the last trimester.

***Socio-Demographic Information Collection Form:*** It is a form prepared by the researcher by scanning the literature, which includes the demographic information, social life characteristics, health habits, and treatments of the women participating in the study.

***The MOS 36 Item Short Form Health Survey:*** The scale (SF 36), which was developed by Ware in 1987 and adapted to the Turkish population after its validity and reliability in Turkish cancer patients by Pinar, 7 was designed to be used in clinical practice and research, evaluation of health policies, and general population studies. 7 8 The scale is universally recognized by this abbreviation, as all studies since its development have used the English abbreviation SF-36 for the scale.

***Center for Epidemiologic Studies Depression Scale:*** The Center for Epidemiologic Studies Depression Scale is a short scale that can be applied to the general population and special groups, designed to measure depressive symptoms. It has been used frequently as a general depression screening tool. The items of the scale consist of symptoms related to depression. The aim of the Epidemiological Research Center Depression Scale is to measure the components that affect the level of current depressive symptoms and depressed mood. The scale has the feature of evaluating the relationship between depression and other variables in subgroups. The Epidemiological Research Center Depression Scale consists of 20 items, each item can receive 0–3 points, and items 4, 8, 12, and 16 are reverse scored. The total score can vary between 0–60, and a score of 16 and above suggests the possibility of depression. The validity and reliability studies in our country were performed by Yilmaz and Beji 9 within the scope of her doctoral thesis and the Cronbach's α reliability coefficient was found to be 0.85 for the whole scale.

***Postpartum Data Collection Form:*** It is a form prepared by the researcher by scanning the literature, which includes the birth type of the women participating in the study, the week of birth, whether they have any postpartum problems, the birth weight of the baby, and the breastfeeding status of the baby.

The data collection tools were applied to the experimental and control group, in the last trimester of pregnancy and in the third month of postpartum, by the method of mutual interview by the researcher. The data obtained in the study were analyzed using SPSS software. Number, percentage, mean, standard deviation, Kolmogorov Smirnov-Normal Distribution, and correlation tests were used for data analysis.

## Results

The mean age of the pregnant women with gestational diabetes was 30 ± 6.79 years, the mean age of healthy pregnant women was 29.03 ± 4.92 years, the year of marriage of pregnant women with gestational diabetes was 7.23 ± 5.23 years, healthy pregnant women was 6.08 ± 3.95 years. There was no statistically significant difference between the two groups in terms of age and year of marriage (Z = -, 938 *p* = 0.348). Women with gestational diabetes were literate and primary school graduates in 16.5 percent ( *n* = 33), middle and high school graduates in 26.5 percent ( *n* = 53), and bachelor's degree holders in 7% ( *n* = 14). 15% of healthy pregnant women ( *n* = 30) were literate and had completed primary school, 25% ( *n* = 50) had completed middle and high school, and 10% ( *n* = 20) had completed a bachelor's degree ( Table 1 ). There was no statistically considerable difference between the two groups in terms of educational level (X 2 = 7.29 *p* = 0.13). When the obstetric characteristics of the pregnant women were examined, pregnant women in the case group the number of pregnancies was 2.67 ± 1.4, the number of optional abortions was 1.43 ± 5.35, the number of abortions was 1.70 ± 0.99, the number of births was 1.66 ± 0.62 and the number of living children was 1.66 ± 0.62. The number of pregnancies in healthy pregnant women was 2.65 ± 1.15, the number of optional abortions was 1.5 ± 0.52, the number of abortions was 1.55 ± 0.69, the number of births was 1.41 ± 0.76 and the number of living children was 1.41 ± 0.76. There was no statistically considerable difference between the two groups in terms of obstetric characteristics ( Table 1 ). It was reported that 36.5% ( *n* = 73) of pregnant women with gestational diabetes and 46% ( *n* = 92) of healthy pregnant women had planned pregnancies ( Table 1 ). When both groups were compared for pregnancy planning, a statistically significant difference was found between them. It was found that healthy pregnant women had a higher rate of pregnancy compared with gestational diabetes (X 2 = 12.50 *p* = 0.000). It was found that 47.5% ( *n* = 95) of pregnant women with gestational diabetes and 48.5% ( *n* = 97) of healthy pregnant women went to regular antenatal controls. There was no statistically considerable difference between the two groups in terms of regular antenatal controls (X 2 = 0.52 *p* = 0.36) ( Table 1 ).

**Table 1:** Obstetric Characteristics of Healthy Pregnant and Pregnant Women with Gestational Diabetes

		Gestational Diabetes Pregnant		Healthy Pregnant		SUM		Z*	P
		X + SS		X + SS					
**Age**		30 ± 6.79		29.03 ± 4.92		29.51 ± 5.93		-0,938	0,348
**Year of Marriage**		7.23 ± 5.23		6.08 ± 3.95		6.66 ± 4.66		-1.061	0,289
**Obstetric** **Features**	Number of Pregnancy	2.67 ± 1.4		2.65 ± 1.15		2.66 ± 1.287		-0,45	0,65
	Optional Abortion Number	1.43 ± ,535		1.5 ± ,522		1.47 ± ,513		-0,29	0,77
	Abortion Number	1.70 ± ,99		1.55 ± ,697		1.61 ± ,897		-0,37	0,704
	Number of Births	1.66 ± ,62		1.41 ± ,757		1.54 ± ,880		-1.48	0,138
	Number of Living Children	1.66 ± ,62		1.41 ± ,757		1.54 ± ,880		-1.48	0,138
		**n**	**%**	**n**	**%**	**n**	**%**	X 2 **	**p**
**Pregnancy Planning Situations**	Yes	73	36,5	92	46	165	82,5	12,50, 000	
	No	27	13,5	8	4	35	17,5		
	Total	100	50	100	50	200	100		
**Regular Antenatal Control**	Yes	95	47,5	97	48,5	192	96	0,52 0,36	
	No	5	2,5	3	1,5	8	4		
	Total	100	50	100	50	200	100		

* Mann-Whitney U test was used.

**X 2 test used

It was found that gestational diabetes was diagnosed at 22 weeks of gestation (22.67 ± 1.5) in pregnant women with gestational diabetes. It was determined that 62% ( *n* = 62) of the pregnant women with gestational diabetes were treated with diet, 36% ( *n* = 36) with insulin therapy, and 2% ( *n* = 2) with both diet and exercise therapy. It was found that the births of pregnant women with gestational diabetes occurred at 37.66 ± 1.49 weeks of gestation and the births of healthy pregnant women at 37.68 ± 1.197 weeks of gestation. 23.5% ( *n* = 47) of pregnant with gestational diabetes had normal births, 26.5% ( *n* = 53) pregnancies ended with cesarean section, 33.5% ( *n* = 67) pregnancies of healthy pregnant women had normal delivery, and 16.5% ( *n* = 33) pregnancies ended with cesarean delivery. It was found that pregnant women with gestational diabetes delivered by cesarean section more than healthy women and there was a statistically significant difference between them ( *p* < .05). It was detected that the mean birth weight of the babies of pregnant women with gestational diabetes was 3451 ± 490 gr, and the mean birth weight of babies of healthy pregnant women was 3459 ± 495 gr. When the breastfeeding status of women was examined, it was seen that all pregnant women with gestational diabetes ( *n* = 100) were breastfeeding, while 99 percent of healthy pregnant women ( *n* = 99) were breastfeeding. It was determined that one of the healthy pregnant women did not breastfeed her baby. It was reported that the reason was that the mother did not breastfeed because her nipple was injured.

Table 2 shows the CESD total score and the average score of the four subscales of gestational diabetes and healthy pregnant women. Gestational diabetic pregnant women and healthy pregnant women during pregnancy, except for the positive emotional sub-size and total scores, there is a statistically significant difference between other subscales. On the other hand, except for depressive, positive sensation, and sub-size of interpersonal relationship, there was a statistically significant difference between other size scores and total scores. When compared with the gestational period and the average of the score of the four subscales of gestational diabetes, it was determined that there was a significant difference between the mean scoring in the scales of depression and depressive sensation from the sub-scales with the total score of depression, positive sensation, interpersonal relations, and physical complaints ( *p* < ,05) ( Table 2 ). During the gestation period and the period of pregnancy of healthy pregnant women, it was not determined that there was only a statistically significant difference between the score averages of the positive emotion subscale (p > , 05). In addition, there was no significant difference between the total score of depression and the mean of depressive sensation from the subscales, interpersonal relations, and physical complaints ( *p* < ,05) ( Table 2 ). When comparing the total score of CESD and the score averages of the four sub-scales of gestational diabetic and healthy pregnant women during pregnancy, there was a significant difference between the mean score in the scales of depressive sensation, interpersonal relations, and bodily complaints ( *p* < 0,05). When the periods of postpartum of both groups were examined, significant headlights were detected between bodily complaints and total scores ( *p* < 0,05) ( Table 2 ).

**Table 2:** Characteristics of subscales of cesd scale in pregnant women with gestational diabetes and healthy pregnant women and comparison of gestational period

	Gestational diabetes pregnant		Healthy pregnant		Healthy pregnant women with gestational diabetes			
	Pregnancy	Postpartum	Pregnancy	Postpartum	Pregnancy		Postpartum	
	X ± SS	X ± SS	X ± SS	X ± SS	Z*	P	Z*	P
Depressed Mood	7.89 ± 3.06	11.06 ± 3.02	7.07 ± 2.29	12.03 ± 3.67	-1.99	0.046	-1.92	0.055
Positive Affect	7.67 ± 1.89	6.99 ± 1.98	7.95 ± 1.36	7.47 ± 2.08	-1.23	0.218	-1.62	0.104
Somatic Complaints	9.21 ± 2.68	12.01 ± 2.49	8.40 ± 2.35	13.11 ± 3.38	-1.97	0.049	-2.32	0.020
Interpersonal Relations	2.09 ± 0.98	2.45 ± 1.05	1.79 ± 0.87	2.69 ± 1.24	-2.40	0.016	-1.09	0.275
Total	26.86 ± 6.12	32.52 ± 5.45	25.21 ± 4.92	35.30 ± 7.97	-1.93	0.053	-2.33	0.020

*Mann-Whitney U test was used

When the total and subscale scores of pregnant women with gestational diabetes and healthy pregnant women were compared with the total and subscale scores of the SF36 scale during pregnancy, there was no statistically notable difference between them ( Table 3 ). When the total and subscale scores of pregnant women with gestational diabetes and healthy pregnant women were compared with the total and subscale scores of the SF36 scale during the period of postpartum, there was no statistically substantial difference between all scales except physical function and pain subscale. Healthy pregnant women scored higher than pregnant women with gestational diabetes in physical function and pain subscale and the difference between them was statistically significant (physical function Z = -2.89 *p* = 0.004 painZ = -2.85 *p* = 0.004) ( Table 3 ).

**Table 3:** Characteristics of Subscales of SF36 Scale in Pregnant Women with Gestational Diabetes and Healthy Pregnant Women

		Pregnancy	Postpartum	Z*	P
		X ± SS	X ± SS		
Gestatıonal diabetes pregnant	Physical Function	54.95 ± 12.88	76.65 ± 9.64	-8,15	0,00
	Physical Function (Role)	18.75 ± 26.44	82.5 ± 27.86	-8,19	0,000
	Emotional Function (Role)	20.66 ± 27.53	76 ± 32.85	-7,55	0,000
	Energy / Fatigue	38.95 ± 13.39	39.2 ± 15.54	-0,22	0,825
	Mental Health	49.92 ± 11.37	47.52 ± 13.70	-0,760	0,447
	Social Function	40 ± 14.93	57.75 ± 14.73	-3,83	0,000
	Ache	35.5 ± 17.196	51 ± 15.142	-5,58	0,000
	General Health	42.25 ± 10.94	42.25 ± 10.94	0,00	1,00
	Health Compared with Last Year	2,51 ± 0,732	3,34 ± 0,742		
Healthy pregnant	Physical Function	52.45 ± 14.48	80.65 ± 9.99	-8,05	0,000
	Physical Function (Role)	16.25 ± 18.59	83.25 ± 24.63	-8,63	0,000
	Emotional Function (Role)	28 ± 29.85	85.33 ± 22.38	-7,54	0,000
	Energy / Fatigue	38.05 ± 11.05	36.15 ± 20.50	-,65	0,516
	Mental Health	51.24 ± 8.78	42.52 ± 17.57	-3,80	0,000
	Social Function	28 ± 29.85	60.12 ± 11.47	-5,54	0,000
	Ache	35.6 ± 18.54	56.6 ± 13.94	-7,16	0,000
	General Health	42.90 ± 11.72	42.90 ± 11.72	0,000	1,00
	Health Compared with Last Year	2,66 ± 0,623	3,47 ± 0,594		

*Mann-Whitney U test was used

When the correlations between SF36 subscales and CESD total scores of pregnant women with gestational diabetes and healthy pregnant women were examined; it was found that only physical function moderate correlation and mental health subscale were found to be highly correlated in gestational diabetes pregnancies during the gestation period. During the postpartum period, there was a weak correlation between physical function, a moderate correlation between physical function, and a high correlation in emotional function subscales. There was a weak correlation between the other subscales. (The correlation between SF36 subscale scores and CESD total scores was found to be r = 0.614 in the gestational period, mental health r = 0.777, the puerperal period in physical function r = 0.538, emotional function r = 0.709) ( Table 4 ). It was found that there was a high correlation between healthy pregnant women in the general health subscale of pregnancy and a weak correlation between the mental health subscale and a very poor correlation between the other subscales. In the postpartum period, it was found that there was a moderate correlation between the social function subscale and a very high correlation between the pain subscale, and a very weak correlation between the other subscales (Correlation between SF36 score subscales and CESD total scores, pregnancy general health r = 0.882 social function r = 0.612 pain r = 0.920) ( Table 4 ).

**Table 4:** Correlation between Pregnancy and Postpartum Period SF 36 Scale Sub-Dimensions and CESD Total Score of Pregnant with Gestational Diabetes and Healthy Pregnancy

		Correlation of SF 36 sub-dimension scores with CESD total score			
		Pregnancy		Postpartum	
		r*	p	r*	p
Gestatıonal diabetes pregnant	Physical Function	0,614	p< 0.005	0,461	p< 0.005
	Physical Function (Role)	0,203	p< 0.005	0,538	p< 0.005
	Emotional Function (Role)	0,357	p< 0.005	0,709	p< 0.005
	Energy / Fatigue	0,217	p< 0.005	0,040	p< 0.005
	Mental Health	0,777	p< 0.005	0,001	p< 0.005
	Social Function	0,351	p< 0.005	0,074	p< 0.005
	Ache	0,016	p< 0.005	0,291	p< 0.005
	General Health	0,185	p< 0.005	0,263	p< 0.005
Healthy pregnant	Physical Function	0,043	p< 0.005	0,006	p< 0.005
	Physical Function (Role)	0,010	p< 0.005	0,001	p< 0.005
	Emotional Function (Role)	0,001	p< 0.005	0,017	p< 0.005
	Energy / Fatigue	0,074	p< 0.005	0,007	p< 0.005
	Mental Health	0,169	p< 0.005	0,000	p< 0.005
	Social Function	0,000	p< 0.005	0,612	p< 0.005
	Ache	0,000	p< 0.005	0,920	p< 0.005
	General Health	0,882	p< 0.005	0,005	p< 0.005

*Pearson Correlation

## Discussion

The mean age of pregnant women with gestational diabetes was the same. The mean of marriage years of pregnant women with gestational diabetes was 7.23 ± 5.23 years, and the mean of marriage years of healthy pregnant women was 6.08 ± 3.95 years. No statistically significant difference was determined between the two groups in terms of age and year of marriage (Z = -,938 *p* = 0.348). In the study performed by Öztanriöver 10 on 46 patients with risky pregnancies and 53 women with no problems in pregnancy, no significant difference was found between the two groups in terms of marriage age (p > 0.05). In the same study, when looking at the risk situations according to the age of marriage, 47 (58%) of pregnant women aged 18 and under carry no risk, 34 (42%) of them carry no risk, 135 (62.5%) of pregnant women aged 19–34 carry no risk, 81 (37.5%) of them carry no risk. There was no statistically significant relationship between the two groups ( *p* = 0.568). Marriage age was found to be ineffective on the risk status of pregnancy. To compare the psychosocial health of risky (More than 4 pregnancies, 35 years or older pregnant women with any systemic disease, hyperemesis gravidarum, urinary system infections, premature birth threat, etc. pregnant women hospitalized for reasons such as) and risk-free pregnant women by Gümüşdaş et al., 2 a significant difference between their age was found in their studies covering 108 risky and 124 risk-free pregnant women. This difference is thought to be owing to the number of women who have a risky pregnancy is 36 years of age and over. 2

When the educational status of pregnant women was examined, there was no statistically significant difference in terms of education levels between gestational diabetes and healthy pregnant women ( *p* = 0.13). Kiliç et al. 11 examined the prevalence of prenatal care and the factors affecting it in pregnant women and puerperal women who completed the 28th gestational week and they did not find any statistical significance between the pregnant women and their education level. In a study conducted by Pesavento et al. 12 on 100 pregnant women with 50 risky and 50 normal pregnancies, to evaluate the quality of life, the presence of depressive symptoms, and their possible relationships in normal and high-risk pregnancies, it was observed that, 6% of risk-free pregnant women and 24% of risk-free pregnant women's education level was lower than secondary school. 58% of risk-free pregnant women and 58% of risk-free pregnant women's education level was secondary school and above. 36% of risk-free pregnant women and 18% of risk-free pregnant women were educated at the university level. 12 In a study conducted by Shawky and Milaat 13 in Saudi Arabia with pregnant women aged 16 and over, it was determined that as the education levels of the pregnant women increased from primary school and lower level to the university level, the risk of pregnancy decreased significantly from a statistical point of view ( *p* < 0.001). 13 In the present study, no significant relationship was found between education levels and risky pregnancies. The results of the above-mentioned studies on this subject are similar to the results of the present study, and only Shawky and Milaat 13 have found that a high educational level reduces the risk of pregnancy. Supportive studies are needed on this subject.

In the present study, a considerable difference was found between delivery methods for gestational diabetes and healthy pregnant women ( *p* < 0.05). It was found that pregnant women with gestational diabetes had a higher delivery type by cesarean section than healthy pregnant women. To determine the quality of life of pregnant women followed up in Italian diabetes clinics, Dalfrà et al. 1 conducted studies on 245 pregnant women (30 of them are type 1 diabetes, 176 of them are gestational diabetes and 39 of them are healthy pregnant women). It was revealed that cesarean rates of pregnant women with gestational diabetes were higher than other pregnant women and show similarity with the present study. 1 The high rate of cesarean section in diabetic pregnant women has negatively affected the quality of life in the puerperium.

CESD total scores and subscale scores of pregnant women with gestational diabetes were found to be statistically higher than the scores of healthy pregnant women at a significant level. Similarly, in the study of Öztanrıöver, 10 the Beck Depression Scale average was found to be significantly higher in risky pregnant women than in risk-free pregnant women ( *p* < 0.001). In the study conducted by Nicholson et al. 14 to determine the independent relationship between health-related quality of life and independent health among various women in the early stages of pregnancy, depressive symptom (CES-D) according to the Center for Epidemiological Studies-Depression (CES-D) scale cut-off point_16) rate (41%) was found to be statistically higher at a significant level ( *p* = 0.01). 14 It is thought that the socio-cultural structure and income status of women who participated in the present study may be effective in showing depressive symptoms both during pregnancy and puerperium.

In a study conducted by Kozhimannil et al. 15 With low-income women to investigate the relationship between diabetes and pregnancy and postpartum depression, depression rates of women with diabetes before gestation and gestational diabetes were found to be higher than those without diabetes.

However, it was found that all women (healthy and with gestational diabetes) in the puerperium had more depressive symptoms than those in the gestational diabetes group. In our study, it was observed that the CESD scores of pregnant women with GDM were higher than those of healthy pregnant women. The reason for this is thought to be due to being diagnosed with diabetes during pregnancy. Although this situation is compatible with the literature, it is seen in the literature that the postpartum depression scores of healthy pregnant women are high. Lack of perceived social support after delivery causes depression in healthy pregnant women. 16 17 To determine the prevalence of postpartum depression, the associated factors, and the effect on the quality of life, Durukan et al. 18 found that the prevalence of postpartum depression was significantly higher and the quality of life scores of mothers with high depression scores were also low. 18 In the study of Atasoy et al., 19 which included 97 women to determine the risk factors that may cause depressive symptoms in the postpartum period, the prevalence of postpartum depression increased in the later weeks of the puerperium and the risk of depression of working mothers was higher. In a study conducted by Minschart et al. 20 to determine the effect of depressive symptoms on pregnancy outcomes and postpartum quality of life in women with gestational diabetes mellitus (GDM) and normal glucose tolerance (NGT), pregnant women with GDM have postpartum depression 1.52 times more often than other pregnant women. was found to be entered. 20 In the study of Tel et al. 21 examining the effects of home visits and planned education on mothers' postpartum depression and quality of life, it was determined that mothers were at risk of depression in the postpartum period and that postpartum depression negatively affected their quality of life. In the study of Do et al. 22 in which they examined postpartum depression and risk factors in Vietnamese women, it was found that the prevalence of postpartum depression in women was 27.6% and GDM was a risk factor for postpartum depression. 22 Our study results are in agreement with the literature.

In the present study, when the total and subscale scores of the SF36 scale of pregnant women were compared, no statistically significant difference was found between them. SF36 scores of healthy pregnant women were found to be higher than those of gestational diabetes. In the study of Dalfrà et al., 1 when the total and lower dimensions of the SF36 scale of pregnant women and healthy pregnant women with gestational diabetes were compared, it was revealed that the role limitation due to physical function, pain, physical problems, and the scores of the lower scales of the general perception of health were found to be statistically different at a significant level, and the other subscales did not have statistical difference at a significant level. 1

In the study conducted by Şahsıvar, 23 to investigate socio-demographic characteristics, depressive symptom levels, quality of life, and their relationship with each other in 297 women with risky and risk-free pregnancies by applying the World Health Organization Quality of Life Scale, it was seen that physical health, psychological health, social relations, and the general area mean scores of the pregnant women in the risk group were lower than those in the risk-free group. The difference between the two groups was statistically significant. 23 In a study conducted by Altiparmak 24 with 259 pregnant women, it was determined that high levels of education and income, social security, and having a nuclear family had an immediate effect on the quality of life and strength of self-care scores and that the scale scores were high to determine the relationship between self-care strength and quality of life.

In the study of Pesavento et al., 12 it was also found that the physical health, psychological health and environmental field scores of at-risk pregnant women were lower than those without risk Drescher et al. 25 aimed to determine the quality of life and functional status of adolescent pregnant women in their study covering 42 adolescent pregnant women. It was observed that there was a significant decrease only in physical health in comparison between the case group (14–18 age group) and control group (18–24 aged pregnant women). 25

In his review of articles on the quality of life in pregnancy and the postpartum period in 2003, Symon 26 found that the concept of quality of life was misused in the literature, thus there were few sources to describe the quality of life in the postpartum care period. However, it was determined that mothers experienced more depressive symptoms, and decreased quality of life in the postpartum period compared with pregnancy periods in the articles examined. 26

In the present study, when the total and subscale scores of the gestational diabetes pregnancies and postpartum SF36 scale were compared, it was found that there was a statistically significant difference between all scales except for the health perception, energy, and mental health subscale. When the total and subscale scores of the healthy pregnant women were compared, it was found that there was a statistically significant difference between all scales except for the health perception and energy subscale. In both groups, it was revealed that the quality-of-life scores increased during the postpartum period compared with the gestational period; however, the total score of SF36 was lower in pregnant women with gestational diabetes compared with healthy pregnant women and their quality of life was affected more negatively.

When a study is cross-sectional, it is limited to patients who were recruited during a specific time period. Furthermore, the study's shortcomings stem from the fact that it was conducted in a single location.

## Conclusion

As a result, it was found that the quality of life of pregnant women with gestational diabetes was lower than healthy pregnant women during the postpartum period. Although the depressive symptoms of all pregnant women were higher in both pregnancy and the postpartum period, it was found that pregnant women diagnosed with gestational diabetes were higher than healthy pregnant women. In light of these results, nurses and other health professionals in pre-pregnancy environments are in a unique position in terms of diagnosing depression during pregnancy and pre-pregnancy. In addition, due to the increased incidence of depression during pregnancy and the adverse effects on the fetus, it is necessary to question the symptoms of depression in routine pregnancy examinations. Women with gestational diabetes should be treated specifically and assessed for both quality of life and depression.
